# Survival in patients receiving reduced dose intensity of bevacizumab for unresectable hepatocellular carcinoma

**DOI:** 10.1038/s41698-025-00908-7

**Published:** 2025-05-06

**Authors:** Dimity Ball, Jean-Charles Nault, Mathew Vithayathil, Manon Allaire, Nathalie Ganne-Carrié, Claudia Campani, Fabio Marra, Rohini Sharma

**Affiliations:** 1 Department of Medical Oncology, Imperial College NHS Healthcare Trust, Hammersmith Hospital, Du Cane Road, W12 0HS London, UK; 2 Centre de recherche des Cordeliers, Sorbonne Université, Inserm, Université Paris Cité, team « Functional Genomics of Solid Tumors », Equipe labellisée Ligue Nationale Contre le Cancer, Labex OncoImmunology, F-75006 Paris, France; 3 3. Liver unit, Avicenne Hospital, APHP, Bobigny, France, University Sorbonne Paris Nord, Bobigny, France; 4 Division of Surgery and Cancer, Imperial College London, Hammersmith Hospital, Du Cane Road, W12 0NN London, UK; 5 Service d’Hépatolo-gastroentérologie, Hôpitaux Universitaires Pitié Salpêtrière - Charles Foix, AP-HP, Sorbonne Université, Paris, France; 6 Dipartimento di Medicina Sperimentale e Clinica, University of Florence, Florence, Italy

**Keywords:** Cancer, Gastrointestinal cancer, Liver cancer, Hepatocellular carcinoma

## Abstract

IMBrave 150 established atezolizumab and bevacizumab as the new standard for advanced hepatocellular carcinoma (HCC) treatment. However, the trial reported significant adverse events leading to bevacizumab dose interruptions or discontinuations. This retrospective, real-world analysis evaluated the effect of reduced bevacizumab dose intensity on clinical outcomes in 354 patients receiving first-line combination immunotherapy for advanced HCC. To minimize immortal time bias, only those on therapy for over 3 months were included. Of 219 patients included in the landmark analysis, 52 received a reduced dose intensity of bevacizumab. The median relative dose intensity (RDTI) of bevacizumab was 75% (range 9.1–96.9%). There was no significant difference in progression-free survival (11.2 vs. 14.8 months, *p* = 0.5) or overall survival (20.4 vs. 26.8 months, *p* = 0.1) between those receiving 100% vs. reduced RDTI. Exploratory analysis showed that even doses under 75% had no survival impact. Treatment-related grade 3/4 adverse events occurred more frequently with RDTI (30.7% vs. 15.5%). Reduced bevacizumab doses do not impact survival.

## Introduction

The introduction of combination immunotherapy with atezolizumab and bevacizumab (A/B) has dramatically changed the outlook for patients with advanced stage hepatocellular cancer (HCC)^1^. The seminal IMBrave150 study reported a median overall survival (OS) of 19.2 months with A/B versus 13.4 months with sorafenib, establishing A/B as the standard of care for the first line management of advanced HCC^2^. Prior to the publication of IMBrave 150, studies of single agent immunotherapy had failed to demonstrate an improvement in OS in either the first- or second-line setting^3,4^.

It is well established the vasculature in HCC is abnormal; following inhibition of VEGF signalling, normalisation of neo-angiogenesis occurs, improving drug delivery and immune surveillance, as well as tumour hypoxia through vascular pruning^5^. Bevacizumab was the first clinically available VEGF inhibitor, and the additive impact of the bevacizumab with atezolizumab in HCC was first recognised in phase 1b GO30140 study which demonstrated significant improvement in progression free survival (PFS) with A/B compared to atezolizumab monotherapy (5.6 vs 3.6 months, hazard ratio (HR) 0.55; 80% CI 0.40–0.74; *p* = 0.01)^6^.

Despite the promise of combination immunotherapy therapy, 17% of patients in IMBRave150 experienced toxicity related to bevacizumab that led to drug interruption and in 14.6% bevacizumab was permanently withdrawn^1^. The impact of either omitting doses or permanently withdrawing bevacizumab in the real-world setting remains unknown. A recent exploratory analysis of IMBrave data to ascertain the impact of halting bevacizumab on clinical outcomes showed no effect on clinical outcomes^7^. However, there are clear additive benefits of VEGF inhibitor to immunotherapy and more data regarding the impact of bevacizumab cessation are required. To take into consideration the impact of both dose reduction and delays, we considered the dose intensity of bevacizumab and correlated this with clinical outcomes in a real-world analysis of patients receiving A/B for advanced stage HCC. To minimise immortal time bias a landmark analyses of OS and PFS was undertaken in those who had received combination therapy for ≥3months comparing those who received reduced dose intensity with those who did not.

## Results

### Baseline characteristics

Of the 354 consecutive patients receiving A/B, eleven patients had never received bevacizumab and were excluded from analysis. We then considered only those patients who had received combination immunotherapy for at least 3months to minimise immortal time bias (*n* = 219). The baseline characteristics of this cohort are shown in Table 1. Majority of patients had Child-Pugh A cirrhosis (83.6%) secondary to viral hepatitis (51.6%). More patients with BCLC B/C disease had received 100% RTDI (*p* = 0.02) compared to reduced RTDI. No other differences were observed in the baseline characteristics (Table 1).

**Table 1:** Baseline characteristics of study population stratified according to RTDI of bevacizumab dose (*N* = 219)

	Total Population	100% RTDI (*n* = 167)	<100% RTDI (*n* = 52)	*p value*
**Median Age (IQR)**	66.1 ± 7	65.1 ± 12.3	67.5 ± 11.2	0.5
**Male Sex**	184 (84.0)	143 (85.6)	41 (78.8)	0.3
**Risk factors for chronic liver disease**				
Non-alcoholic fatty liver disease	78 (35.6)	58 (34.7)	20 (38.4)	0.7
Alcohol related	98 (44.7)	74 (44.3)	23 (44.2)	1.0
Hepatitis B infection	39 (17.8)	34 (30.3)	5 (9.6)	0.1
Hepatitis C infection	74 (33.8)	57 (34.1)	17 (32.7)	1.0
Other	5 (2.7)	4 (2.4)	1 (1.9)	1.0
**Child-Turcotte-Pugh class**				
A	183 (83.6)	136 (81.4)	47 (90.4)	0.06
B	27 (12.3)	27 (16.2)	0(0)	
**Portal vein thrombus (PVT)**				
Present	77 (35.2)	57 (34.1)	20 (38.4)	0.6
**Extrahepatic spread (EHS)**				
Present	58 (26.5)	48 (28.7)	10 (19.2)	0.2
**Alpha-fetoprotein level (AFP)**				
<400 µg/L	145 (66.2)	111 (66.5)	34 (65.4)	0.7
>400 µg/L	74 (33.8)	56 (33.5)	18 (34.6)	
**ECOG-PS**				
0	87 (39.7)	69 (41.3)	18 (34.6)	0.7
1	104 (47.4)	81 (48.5)	23 (44.2)	
2	3 (1.4)	2 (1.2)	1 (1.9)	
**Barcelona Clinic Liver Cancer Stage**				
**A**	6 (2.7)	2 (1.2)	4 (7.7)	**0.02**
**B**	79 (36.1)	59 (35.5)	20 (38.4)	
**C**	133 (60.74)	106 (63.5)	27 (51.9)	

**Notes**: n (%) for discrete variables; mean ± standard deviation for continuous variables.

*AFP* alpha-fetoprotein, ECOG-PS Eastern Cooperative Oncology Group Performance Status.

Bold indicates significance, *P* < 0.05.

The median PTDI of bevacizumab of the entire cohort was 375 mg/week (range 210 mg/week – 643.5 mg/week) while the median ATDI received was 355 mg/week (range 42.3 mg/week – 643.5 mg/week). The mean RTDI of bevacizumab received was 92.7% (9.1% - 100%). All patients received the PTDI of atezolizumab: 400 mg/week, median RTDI 100%. When considering those patients who received a reduced dose intensity of bevacizumab, the median RTDI received was 75% (range 9.1–96.9%). Of the patients who received a reduced RTDI, 17 (10%) missed one treatment cycle, 14 (8.3%) missed 2 or more and 19 (11.4%) permanently discontinued bevacizumab and continued on atezolizumab alone. The commonest causes of bevacizumab discontinuation were bleeding events (20.5%) (Table 2) and hepatic decompensation (4%). No patient discontinued bevacizumab for reasons other than adverse events. The commonest cause for temporary interruption to bevacizumab dosing included hypertension (*n* = 9) and bleeding from any site (*n* = 8). Other reasons for temporary interruption of bevacizumab unrelated to toxicities experienced included reduced performance status (*n* = 2) and procedures (*n* = 3).

**Table 2:** Adverse events experienced by those receiving full dose (*n* = 167) and reduced dose intensity of bevacizumab (*n* = 52)

Events secondary to bevacizumab	Full Dose Intensity (*n* = 167)		Reduced Dose Intensity (n = 52)	
	Any Grade no. (%)	G3/4 no. (%)	Any Grade no. (%)	G3/4 no. (%)
Fatigue	61 (37)	0 (0)	22 (42)	1 (2)
Hypertension	26 (16)	7 (4)	11 (21)	4 (8)
Bleeding other sites*	22 (13)	0 (0)	8 (16)	0 (0)
Proteinuria	10 (6)	4 (2)	10 (19)	1 (2)
Variceal bleeding	6 (4)	4 (2)	8 (15)	6 (12)
Mucositis	5 (3)	0 (0)	8 (15)	1 (2)
Cardiac disease	2 (1)	0 (0)	0 (0)	0 (0)
Thrombosis	1 (0.6)	0 (0)	1 (2)	0 (0)
**Events secondary to atezolizumab**				
MSK disorders	22 (13)	0 (0)	6 (12)	1 (2)
Pruritis	27 (16)	0 (0)	8 (15)	1 (2)
Rash	19 (11)	3 (2)	8 (15)	0 (0)
Diarrhoea	16 (10)	4 (2)	4 (8)	0 (0)
ALT derangement	10 (6)	1 (0.6)	3 (6)	0 (0)
Hypothyroidism	10 (6)	0 (0)	3 (6)	0 (0)
Hyperthyroidism	7 (4)	1 (0.6)	1 (2)	0 (0)
Hepatitis	4 (2)	1 (0.6)	1 (2)	1 (2)
Respiratory disorder	2 (1)	0 (0)	0 (0)	0 (0)
Pancreatitis	1 (0.6)	0 (0)	0 (0)	0 (0)
Pituitary dysfunction	0 (0)	0 (0)	1 (2)	0 (0)
Adrenal dysfunction	0 (0)	0 (0)	0 (0)	0 (0)

Adverse events graded according to NCI-CTC version 50.

ALT alanine transaminase, MSK musculoskeletal.

* Bleeding from other sites including epistaxis (*N* = 22), haemoptysis (*N* = 2), rectal bleeding (*N* = 2), intracranial haemorrhage (*N* = 2), haematuria (*N* = 1), retinal haemorrhage (*N* = 1).

### Efficacy analysis

**Fig. 1:**
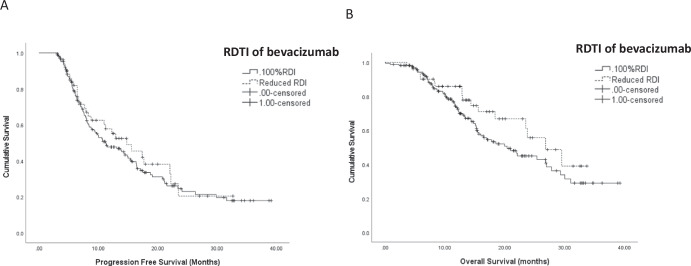
Survival curves illustrating the effect of RDTI of bevacizumab. **A** Kaplan Meier analysis of RDTI of bevacizumab received and progression free survival (months). **B** Kaplan Meier analysis of RDTI of bevacizumab received and overall survival (months).

At the data cut-off of December 2023, the median follow-up duration was 14.7 months (range 0.4–39.3months). The PFS and OS for the entire study population was 12.2 months (95% CI: 9.1–15.5) and 23.5moths (95% CI: 17.2–29.9), respectively. We considered the impact of RDTI of bevacizumab on survival outcomes to combination immunotherapy. In terms PFS, no significant difference was observed in those patients receiving a bevacizumab RDTI < 100% (14.8 months, 95% CI: 8.9–20.7) compared to those who received an RDTI of 100% (11.2 months, 95% CI: 7.8–14.5, *p* = 0.5) (Fig. 1A). Similarly, when considering OS, no difference was observed between patients receiving an RDTI of bevacizumab <100% (26.8 months, 95% CI: 19.6–34.1) compared to those receiving RDTI 100% (20.4 months, 95% CI 15.3–25.5, *p* = 0.1)(Fig. 1B). In order to identify and adjust for potential confounding factors we conducted a multivariable analysis of features known to influence outcome to combination immunotherapy including BCLC stage, PVT and extrahepatic spread. RDTI of bevacizumab remained not predictive of either PFS and OS outcome when taking into consideration these factors (supplementary Table 1 and 2). We then considered only those patients who received a reduced dose intensity of bevacizumab. Taking the median RDTI of 75%, we investigated the impact of this on both PFS and OS in order to ascertain if a 25% reduction or more in dose intensity had an impact on outcome. Similar to the results from the entire cohort we noted no difference in the PFS between those receiving less than or greater than 75%RDTI (HR 1.1, 95% CF: 0.5–2.3, *p* = 0.8) and OS (HR 0.9, 95%CI: 0.3 ± 2.3, *p* = 0.8).

In sensitivity analysis, no difference was observed for PFS (RTDI 100%: median PFS 16.4 months, 95% CI 10.0–20.8 vs. RTDI < 100%: median PFS 14.8months, 95% CI 8.3–22.1) and OS (RTDI 100%: median OS 22.1months, 95% CI 17.0-inf vs. RTDI < 100%: median OS 26.8months, 95% CI 18.4-inf), for bevacizumab RDTI propensity score matched groups, based on baseline clinical variables.

### Safety

When considering the entire treatment cohort, any grade trAE occurred 70.7% in the full dose group and 84.6% in the reduced dose group (Table 2). Grade 3/4 trAEs occurred in 26 (15.5%) of patients receiving 100% RDTI of bevacizumab while 16 (30.7%) of those receiving a reduced dose intensity experienced grade 3/4 toxicity. No grade 5 trAEs were recorded in either patient group. We then considered trAEs directly attributable to bevacizumab. The commonest bevacizumab trAEs occurring in more than 10% of patients were fatigue reported in 37% of patients receiving full dose bevacizumab and 42% receiving reduced dose intensity, and hypertension occurring in 16% and 21%, respectively (Table 2). Bleeding from any site occurred in 13% of those receiving full dose bevacizumab and 16% of those who received a reduced dose. Bleeding from any site resulted in bevacizumab interruption or withdrawal in 21% of all patients. proteinuria of any grade resulted in bevacizumab interruption or withdrawal in 17% and hypertension of any grade in 10%. The median time to interruption of bevacizumab due to toxicity was 6.0months (IQR 4.4 months) while the median time to cessation due to toxicity was 6.3months (IQR 7.35months).

## Discussion

In a prospective, real-world study we conducted a land-mark analysis that has shown that a reduced dose intensity of bevacizumab has no impact on PFS or OS. Overall, patients received 20 mg/week less of bevacizumab which did not impact on survival outcomes. Furthermore, in a subgroup of all patients who had experienced a dose delay or discontinuation, we observed no difference in outcome for those receiving greater or less than 75% RDTI. These findings are consistent with the previous subgroup analysis of the IMBrave 150 and the GO30140 studies^7,8^. However, unlike these studies that only report the impact of omitting doses, we have considered the impact of dose intensity on treatment outcome which objectively quantifies dose received over unit time which accounts for dose delays, reductions and omissions. Moreover, we have considered the impact of bevacizumab interruption from all causes not just trAEs.

Conflicting results were reported by a recent real-world study conducted in Japan considering the impact of bevacizumab on clinical outcomes^9^. In the landmark analysis by Hatanaka and colleagues, which considered the impact of bevacizumab interruption within the first 9 weeks of therapy, the authors report a significant negative impact on both PFS and OS. However, in this almost 50% of patients had received previous lines of therapy while our analysis only includes those who received first-line systemic therapy. Moreover, the study by Hatanaka and colleagues focuses on the impact of early interruption. Only 18 patients in our cohort experienced bevacizumab related toxicity requiring dose interruption or cessation within the first 9 weeks of therapy which may relate to differences in the patient demographics as outlined. We performed a landmark analysis at 3months as the majority of trAEs that resulted in reduced bevacizumab dosing in the IMBrave 150 study occurred between 2-3months^7^. Moreover, we reported the impact of bevacizumab interruption from any cause not just from AEs such that this data is more applicable to the clinical setting where factors such a delayed wound healing, drug availability or scheduling may occur.

The optimal dose of bevacizumab required to achieve maximal clinical benefit while avoiding adverse events remains unclear^10^. The seminal IMBrave 150 study used 15 mg/kg every 3 weeks and 17% of patients experienced bevacizumab related adverse events that necessitated dose interruption suggesting that there is capacity to reduce dose intensity of bevacizumab while retaining clinical benefit^11^. In the early dose finding studies of bevacizumab, responses were observed with doses as low as 0.3 mg/kg suggesting that the biologic effects of bevacizumab may occur at doses much lower than those clinically used^12^. This may explain the equivalent survival outcomes in our work even with RDTI < 75%. A study in metastatic colorectal cancer compared 5 mg/kg 2weekly with 10 mg/kg 2 weekly combined with irinotecan and reported no difference in clinical outcome with the lower dosing regimen^13^. A further study in HCC investigated 5 mg/kg or 10 mg/kg every 2 weeks and illustrated no difference in efficacy although the study was underpowered to address the specific question of dose^14^. There is no clear dose-response relationship with anti-angiogenics, and as with many biologic agents, the use of maximal tolerated dose as an endpoint in early phase studies is not satisfactory given the lack of toxicity.

There is also an emerging therapeutic concern that too high a dose of anti-VEGF therapy may be detrimental to the tumour microenvironment^15^. When first introduced, the premise of anti-angiogenic therapy was to inhibit new vessel formation and destroy tumour feeding vasculature with a view to reduce nutrients and oxygen to the tumour resulting in subsequent cancer death. However, preclinical work suggests that complete blockade of angiogenesis with high doses of anti-VEGF drugs may increase tumoral hypoxia and create an immunosuppressive environment within the tumour mediated by Ly6C^low^ monocytes and Ly6G^+^ neutrophils^16–18^. While lower doses of anti-VEGF therapy in vivo results in vessel normalisation, improved tumour perfusion and the promotion infiltration of CD8^+^ and CD4^+^ T cells restoring an immunosupportive environment^16^. There is a need for further preclinical work, particularly in HCC, focussing on dose response with regards to the changes in the immune infiltrate with vascular normalisation and destruction.

The issue of dose is important and not limited to bevacizumab and can be extended to immunotherapy. This is of particular interest given the cost for healthcare systems both in terms of managing toxicities and the cost of the drugs themselves^19^. The issue of dose frequency is currently being addressed in lung cancer through a prospective study considering 6weekly versus 12weekly schedules for pembrolizumab (NCT05085028), and we suggest that a similar study would of great utility in HCC particularly with regards to the frequency of atezolizumab.

This study suffers from the limitations of real-world studies being non-randomised and exploratory in nature, particularly given the small sample size. Nevertheless, this study lends further evidence that the RTDI of bevacizumab can be safely reduced without impacting clinical outcome, reassuring clinicians.

## Conclusions

In this landmark analysis, we have illustrated that a reduced RDTI of bevacizumab from any cause does not have a negative impact on survival outcomes. Findings should be prospectively validated and may pave the way for a reduced dosing schedule saving money for healthcare systems.

## Methods

### Study participants and design

This was a multi-centre retrospective cohort study. Study participants were consecutive patients with unresectable HCC receiving A/B across two tertiary centres in France (Avicenne *n* = 154 and Pitié Salpetrière Hospital *n* = 107), and one centre in Italy (Florence, *n* = 41) and United Kingdom (*n* = 52) recruited between April 2020 and December 2023. All patients had a histological or radiological diagnosis of HCC in accordance with either European Association for Study of the Liver (EASL) or American Association for the Study of Liver Diseases (AASLD) criteria^20,21^ and were deemed suitable for A/B following local multidisciplinary meeting discussion. Patients receiving previous systemic cancer therapy were excluded. All patients received combination A/B in accordance with the IMbrave150 protocol: atezolizumab 1200 mg and bevacizumab 15 mg/kg intravenously every 3 weeks^22^. Dosing modification and toxicity management were conducted by local treating teams. Decisions for treatment discontinuation due to disease progression and/or unacceptable toxicity was made by multi-disciplinary assessment at each local centre.

### Patient outcomes

Patients’ baseline demographics and clinical parameters including underlying liver disease aetiology, Child-Pugh class, Barcelona Clinic Liver Class (BCLC) stage, Eastern Cooperative Oncology Group Performance Status (ECOG-PS) were collected. Overall survival (OS) was defined as the time in months from first drug administration to date of death or date of last follow-up. Progression-free survival (PFS) was the time from first drug administration to date of progression on radiological imaging or death whichever came first. Treatment response was evaluated using RECIST criteria v1.1^23^ on CT or MRI at 9-12 week intervals. Treatment-related adverse events (trAEs) for bevacizumab were defined as per the summary of products characteristics (SmPC). TrAEs were graded as per the National Cancer Institute Common Terminology Criteria for Adverse Events (CTCAE) v. 5.0.

Planned total dose intensity (PTDI) was the planned dose intensity over the entire treatment duration, averaged across the agents used. In case of permanent treatment discontinuation, other than disease progression (PD) or death, the remaining cycles were calculated with the planned length and zero dose. For patients who withdrew from treatment due to PD or death the PTDI was calculated based on the number of cycles actually completed.

$$\mathrm{PTDI}\,(\mathrm{mg}/\mathrm{week})=\frac{\mathrm{Actual\; total\; dose}\,(\mathrm{mg})}{\mathrm{Duration\; of\; therapy}\,(\mathrm{weeks})}$$

Actual total dose intensity (ATDI) was defined as the actual average dose intensity over the real treatment duration.

$$\mathrm{ATDI}\,(\mathrm{mg}/\mathrm{week})=\frac{\mathrm{Actual\; total\; dose}\,(\mathrm{mg})}{\mathrm{Duration\; of\; therapy}\,(\mathrm{weeks})}$$

Relative Total Dose Intensity (RTDI) was defined as the ratio of Actual Total Dose Intensity (ATDI) and Planned Total Dose Intensity (PTDI), expressed as a percentage. RTDI expresses the effect of reductions, delays as well as premature discontinuations (due to the reasons other than PD or death).

$$\mathrm{RTDI}( \% )=\frac{\mathrm{ATDI}}{\mathrm{ATDI}}\,{\rm{x}}\,100$$

### Statistical analysis

To minimise immortal time bias a landmark analyses of OS and PFS was performed in patients who received A/B for ≥3months, including those who experienced the first instance of PD within the first 3 months of treatment. The 3 month landmark was chosen as this was the median time for bevacizumab trAE^24^. For analysis, patients were divided into two cohorts according to whether they received RTDI of 100% or less. Baseline characteristics were compared between the two groups. *χ*^2^ test was used to compare categorical data, and unpaired student *t* test for continuous data.

Time-to-event analysis was performed using Kaplan–Meier method. OS and PFS were compared between both cohorts using log-rank. A *p* value of less than 0.05 was defined as statistically significant. To examine the effect of baseline clinical parameters in normal and reduced bevacizumab intensity groups, a sub-analysis with propensity score matching was performed, using the nearest neighbour matching algorithm. Baseline characteristics including Child-Pugh class, BCLC stage, PVT, extrahepatic spread, liver disease aetiology, ECOG-PS and number of tumour nodules were used for propensity score matching. OS and PFS was analysed based on propensity score matched RTDI groups using Kaplan-Meier analysis. This study received ethical approval by Sheffield Ethics Committee (IRAS 198951), and by the local ethics committee of each treating centre (Groupe Hospitalier Pitié-Salpétrière : APHP210845 / N° IDRCB: 2021-A01065-36, Service d’hépatologie Hôpital Avicenne: CLEA-2023-n°309, University of Florence: CEAVC 14555). The study was conducted in accordance with Good Clinical Practice standards and in respect of the ethical framework stipulated within the Declaration of Helsinki.

## Supplementary information

Supplementary information

## Data Availability

No datasets were generated or analysed during the current study.
