# Supplementary material for: Survival in patients receiving reduced dose intensity of bevacizumab for unresectable hepatocellular carcinoma
Source: NPJ Precis Oncol. 2025 May 6;9:129. doi: 10.1038/s41698-025-00908-7 (PMC12056213; doi:10.1038/s41698-025-00908-7)
Supplement: Supplementary file 1 — Supplementary information [file 41698_2025_908_MOESM1_ESM.docx]

| **Univariate analysis** | | | | **Multivariate analysis** | | | |
| --- | --- | --- | --- | --- | --- | --- | --- |
| **Factor** | **HR** | **CI** | **P value** | **Factor** | **HR** | **CI** | **P value** |
| BCLC stage | 1.3 | 0.9-1.8 | 0.2 | BCLC stage | 1.4 | 0.9-2.2 | 0.2 |
| PVT | 0.9 | 0.6-1.3 | 0.5 | PVT | 0.8 | 0.5-1.2 | 0.3 |
| EHS | 1.3 | 0.9-1.9 | 0.1 | EHS | 1.1 | 0.7-1.7 | 0.8 |
| Bevacizumab RDI | 0.9 | 0.6-1.3 | 0.5 | Bevacizumab RDI | 1.0 | 0.6-1.5 | 0.9 |

**Supplementary table 1. Univariate and multivariate analysis of progression free survival**

BCLC- Barcelona Clinic Liver Class, PVT – portal vein thrombosis, EHS – extrahepatic spread, RDI – relative dose intensity

| **Univariate analysis** | | | | **Multivariate analysis** | | | |
| --- | --- | --- | --- | --- | --- | --- | --- |
| **Factor** | **HR** | **CI** | **P value** | **Factor** | **HR** | **CI** | **P value** |
| BCLC stage | 1.0 | 0.6-1.4 | 0.8 | BCLC stage | 1.0 | 0.6-1.9 | 0.9 |
| PVT | 0.8 | 0.5-1.2 | 0.3 | PVT | 0.8 | 0.4-1.4 | 0.4 |
| EHS | 1.0 | 0.6-1.6 | 0.9 | EHS | 0.9 | 0.5-1.6 | 0.6 |
| Bevacizumab RDI | 0.7 | 0.4-1.1 | 0.1 | Bevacizumab RDI | 0.7 | 0.4-1.2 | 0.2 |

**Supplementary table 2. Univariate and multivariate analysis of overall survival**

BCLC- Barcelona Clinic Liver Class, PVT – portal vein thrombosis, EHS – extrahepatic spread, RDI – relative dose intensity
